# High-Throughput Proteomics Sample Preparation Using a 96-Channel Pipettor and Magnetic Pin Device

**DOI:** 10.1021/acs.jproteome.5c01020

**Published:** 2026-02-16

**Authors:** Georgia Roumelioti, Alex Montoya, Gemma L. M. Fisher, Eneko Pascual Navarro, Angela Woods, Jane Bennett, Naveenan Navaratnam, Oliver Gonzalez-Carvajal, Jodie Birch, Elizabeth Pyman, Sijia Yu, Aleksandra Gruevska, Luc-Alban Vuillemenot, Oleh Lushchak, Zoe Hall, Alexis R. Barr, Christian Speck, Santiago Vernia, William R. Scott, Jesus Gil, Luis Aragon, Louise Fets, David Carling, Pavel V. Shliaha

**Affiliations:** † 47697 MRC Laboratory of Medical Sciences (LMS), London W12 0HS, U.K.; ‡ Institute of Clinical Sciences, 4615 Imperial College London, Hammersmith Hospital Campus, London W12 0HS, U.K.; § Department of Metabolism, Digestion and Reproduction, Imperial College London, Hammersmith Hospital Campus, London W12 0NN, U.K.; 4 Kyiv School of Economics, Kyiv 02000, Ukraine; 5 Biomedical Research Center, Carpathian National University, Ivano-Frankivsk 76018, Ukraine; 6 Institute of Biomedicine of Valencia (IBV), CSIC, Valencia 46012, Spain; 7 Valencia Biomedical Research Foundation, Centro de Investigación Príncipe Felipe (CIPF) - Associated Unit to the IBV, CSIC, Valencia 46012, Spain

**Keywords:** proteomics, phosphoproteomics, high-throughput sample preparation, oasis HLB, SP3, PAC

## Abstract

High-throughput proteomics requires efficient and highly reproducible sample processing, yet workflows—particularly for PTM profiling—remain complex and costly to fully automate. Here, we present a practical intermediate solution using manually operated 96-channel devices: the Gilson Platemaster P220 pipettor and VP Scientific 96-well magnetic pin device. Using this setup, we achieved robust and reproducible phosphoproteomics in a 96-well format, completing protein aggregation capture (PAC/SP3) digestion, desalting, phosphopeptide enrichment, and a second desalting step within 2 days while minimizing operator workload and variability. Several innovations enable this workflow. First, we describe a cost-efficient method to generate 96-well solid-phase extraction plates by directly packing the Oasis HLB sorbent into tapered filter plates. We extensively characterize these plates in terms of loading capacity, lipid removal efficiency, and suitability for high-pH fractionation. Second, we demonstrate that efficient PAC digestion does not require continuous bead suspension; instead, digestion can be achieved by briefly aspirating beads in protease solution, eliminating the need for orbital shaking and simplifying automation. The presented workflow familiarizes users with 96-channel devices and hence serves as a good step toward full automation.

## Graphical abstract

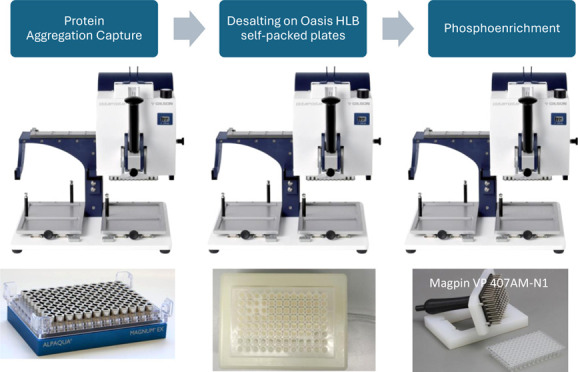

## Introduction

The field of mass spectrometry-based proteomics is undergoing major expansion, driven by recently released platforms that enable high-throughput sample analysis.^1–3^ These instruments can quantify thousands of proteins within minutes,^4^ and complete coverage of the human proteome has recently been reported in as little as 1 h.^5^

The significant increase in liquid chromatography–mass spectrometry (LC–MS) analysis capabilities necessitates a corresponding enhancement in sample processing capabilities. Generally, any bottom-up proteomics experiment begins with the samples, where proteins are embedded in complex environments such as cells, biofluids, or culture media and concludes with clean peptides ready for LC–MS analysis. Therefore, any sample preparation method involves several crucial stages: protein extraction and solubilization, reduction and alkylation to block cysteines, digestion, and the removal of solubilization reagents. In practice, these steps can typically be achieved in three different ways.^6^

The first method involves directly digesting the protein, followed by purification at the peptide level by solid-phase extraction (SPE). This approach is compatible with extraction reagents that can be removed by SPE, such as chaotropic agents or organic solvents like trifluoroethanol.^7^ After SPE, the organic peptide eluate is dried and then resuspended in an aqueous solution to facilitate binding to the reverse-phase LC column. However, this desalting step can be avoided by loading the sample directly onto a disposable precolumn, as recently introduced in the evoSep system.^8^

The second method is protein precipitation,^9^ followed by the digestion of the pellet, producing a cleaned digest ready for LC–MS analysis.^10^ Classical protein precipitation methods, such as chloroform–methanol, are effective for cleanup but are not suitable for reproducible large-scale sample preparation. Therefore, Hughes et al. suggested a single-pot, solid-phase-enhanced sample preparation (SP3) technique, whereby proteins are precipitated on the surface of magnetic beads, facilitating manipulation of the protein pellet by magnetic devices.^11^ They hypothesized that there was a type of chromatographic interaction between the magnetic carboxylate beads and the precipitated protein, but later investigations demonstrated that this interaction is not truly chromatographic, as it can be performed with beads of any chemistry.^12^ Consequently, the authors named the approach protein aggregation capture (PAC), with PAC and SP3 terms now used interchangeably. Another method to capture the protein suspension is to use glass fiber, a process known as suspension trapping (S-Trap).^13^ The strength of precipitation methods lies in their compatibility with almost any reagent, particularly detergents that are especially favorable for protein extraction. However, a significant drawback is that proteins under 10 kDa are not effectively precipitated.^14^

The third method, known as filter-aided sample preparation (FASP), is compatible with protein extraction in any buffer. Contaminants are removed through a buffer exchange using a molecular weight cutoff (MWCO) filter, while proteins are retained and digested on the filter. The resulting clean peptides then pass through the MWCO filter.^15^

Sielaff et al. compared the iST (SPE), FASP, and PAC methods,^16^ while more recently, Varnavides et al. conducted an impressively systematic study of 16 different sample preparation methods that represent these three principal workflows.^17^ Their broad conclusion indicates that all the methods provide comparable protein coverage and can be successfully used in proteomics. However, they caution against using FASP for low-input samples (<20 μg) due to the absorption of material by the MWCO membrane.

All three of these approaches can and have been automated. In a review, Van Eyk et al. summarize their own efforts and those of others, focusing on the in-solution digestion of plasma samples.^6^ Initially, they implemented an in-solution digest with nondesalted samples loaded directly onto the column.^18^ However, their later work has incorporated desalting steps using the Oasis HLB material.^19^ The group of inventors behind SP3 has published an automation method using the Agilent Bravo system, demonstrating consistent performance for low-input samples and FFPE tissues.^20^ Wu et al. likewise automated the thermal proteome profiling on the Agilent Bravo system.^21^ The Thermo KingFisher platform is a dedicated bead-manipulation instrument that transfers magnetic beads via rods and disposable combs; it is popular in PAC and phosphopeptide enrichment workflows but is not a full liquid handler and therefore cannot perform steps that rely on reagent addition.^22^ The Opentrons OT-2 offers a low-cost, open-source liquid handler equipped with a magnetic module. Various groups have successfully implemented PAC on the OT-2 in combination with several other tasks, including concentration adjustment,^23^ TMT labeling,^24^ loading evotips,^25^ and even single-cell proteomics.^26^ Additionally, FASP can also be automated using a positive pressure unit. However, preparing low amounts of protein (<36 μg) can lead to losses due to membrane adsorption.^27^ Notably, none of these automation approaches describes an end-to-end solution suitable for phosphoproteomics. Such a solution would require a pipetting system, magnetic module, heater–shaker, spectrophotometer, and a positive pressure module on deck. The only publication describing such a system to our knowledge has recently been released by the Biognosys team.^28^

Initially, when we began executing proteomics and phosphoproteomics in a 96-well format, we used 12-channel pipettes. However, we quickly realized that the necessity to change tips between samples resulted in excessive waste of plastic and significant time spent on replacing tips. This observation led us to the idea of a transition to 96-channel devices. Müller et al. highlight an important aspect of 96-channel liquid-handling devices: each tip is dedicated to a single sample.^20^ This eliminates cross-contamination and, hence, enables the same tips to be reused throughout a workflow. As a result, plastic consumption is dramatically reduced, and processing times are shortened.

We leveraged this principle to develop a new phosphoproteomics workflow using recently released manual 96-channel tools: the Gilson PlateMaster P220 and the VP Scientific MagPin device. We describe a full 96-well phosphoproteomics experiment, including PAC digestion, desalting, phosphopeptide enrichment, and a second desalting step, within just 2 days. Working in a 96-well format minimizes operator workload and eliminates the possibility of sample mix-up. Moreover, the entire system can be assembled for under 20,000 $ and the protocols can be executed by almost any laboratory technician without the need for specialized training in liquid-handling programming.

## Methods

### Cell Culture

THP-1 cells (ATCC) were cultured at 37 °C in 5% CO_2_ RPMI 1640 medium supplemented with GlutaMAX (2 mM), sodium pyruvate (1 mM), 10% heat-inactivated FBS, and 1× Penicillin/Streptomycin (all obtained from Thermo Fisher Scientific). Twelve liters of *Escherichia coli* (strain DH5α (NEB, C2987) were grown from a single colony at 37 °C with 200 rpm shaking in lysogeny broth. Cells were harvested by centrifugation (4 °C, 20 min at 4000*g*) at mid log-phase. Twelve liters of *Saccharomyces cerevisiae* (strain ATCC 204508/S288C) were grown in yeast peptone media supplemented with 2% glucose and 40 mg/mL adenine. Cultures were seeded at an OD_600_ of 0.2, grown at 30 °C with 180 rpm shaking, and harvested at an OD_600_ of 1.0 by centrifugation (4 °C, 15 min at 3500 g). All cell pellets were washed three times with PBS.

### Protein Extraction

For the three-proteome mix experiment, we needed to get each of the proteomes in the same surfactant cocktail (SEPOD buffer) described by Shen et al.^29^ prior to mixing them at the required ratios. To prepare *E. coli*, a total of 1200 OD_600_ units were lysed in 25 mL of SEPOD buffer using a Branson sonicator set to 10% power. The sonication cycle consisted of 10 s on and 30 s off. For *S. cerevisiae*, 4000 OD_600_ units were extracted using a modified procedure from von der Haar, with β-mercaptoethanol substituted by 20 mM TCEP, and high pH treatment limited to 1 min.^30^ After extraction, *S. cerevisiae* lysates were precipitated in 80% acetone and then resuspended in SEPOD buffer with a Branson sonicator at 10% strength, with a cycle of 10 s on and 30 s off. For the THP-1 samples, 320 million cells were lysed in 10 mL of SEPOD buffer, with 10 μL of benzonase (from a 250 U/μL stock). The mixture was kept on ice for 15 min to ensure complete digestion of DNA, followed by shaking and heating at 70 °C for 30 min, which resulted in sample clearing. This lysis protocol achieved a recovery of approximately 5 g/L from each organism, as measured by the BCA assay. The recoveries were 130 μg/OD_600_ unit for *E. coli*, 90 μg/OD_600_ unit for *S. cerevisiae*, and 150 μg per million THP-1 cells. Prior to digestion, the sample concentrations were adjusted to 2 g/L.

### Precipitation-Assisted Capture

Sample preparation for either THP-1 or a three-proteome mix was performed as follows. Samples were diluted to a 2 g/L concentration, and 150 μL of the sample corresponding to 300 μg of protein was mixed with 150 μL of a solution of 40 mM CAA/20 mM TCEP, incubated at 37 °C for 30 min, achieving simultaneous reduction, alkylation, and dilution necessary for efficient precipitation.^29^ Proteins were precipitated on 1500 μg of hydroxyl beads (MR-HYX010) with precipitation carried out in 60% ethanol (300 μL of sample + 75 μL of bead solution + 560 μL of ethanol) followed by three washes in 80% ethanol.^31^ Samples were digested overnight in 300 μL of 50 mM ammonium bicarbonate with trypsin and LysC at a 1:50, 1:100 ratio, respectively. After protease addition, the beads were pipetted up and down for 2 min and then allowed to settle overnight for digestion at 37 °C. After digestion, the samples were moved to a new plate, the beads were washed by pipetting for 1 min in 150 μL of 0.1% TFA, and the wash was added to the lysate, resulting in around 450 μL of digest. We set aside 50 μL for the analysis of unmodified peptides and subjected the rest to desalting.

### Desalting on Oasis HLB Plates

Desalting was conducted following the manufacturer’s recommendations for the Oasis HLB material (Waters), adjusting wash buffer volumes for the corresponding amount of packed material. For desalting of 300 μg of material, we used 30 mg of the Oasis HLB beads. After loading, peptides were washed 3 times with 500 μL of 0.1% TFA and eluted in 480 μL of ACN and 81 μL of 0.1% TFA to generate a final volume of 600 μL of eluate with 80% ACN. Washing steps were performed using a vacuum manifold by adding solutions directly on top of the beads. For the loading and elution steps, we pipetted the beads up and down in the respective solutions. This batch-mode chromatography approach ensures maximum recovery and makes the process independent of solvent flow rates across the individual wells. Peptides were eluted by centrifugation, which was not performed at a single rcf, but the speed was ramped up to 300 g over 5 min for additional elution in column mode. Ramping rcf ensured that even if the wells exhibited different backpressures, there would still be a reasonable flow rate through the beads at a certain point in the centrifugation ramp.

### Phosphoproteome Enrichment

The phosphoenrichment procedure was performed using Zr-IMAC HP beads (resynbio) as recommended by the manufacturer. After desalting, peptides were eluted in 600 μL of 80% ACN into a deep-well plate (EP0030508203). We then added 30 μL of 5% TFA and 12 μL of 5 M glycolic acid to the samples in a single step to achieve the desired initial buffer composition. After binding for 20 min with continuous shaking at 1200 rpm, the flow-through solution was removed, 150 μL of loading buffer was added to the beads, and the suspension was transferred to Corning 3365 plates. From this point on, the bead transfers between the wash solutions were performed with the MagPin device (VP 407AM-N1, V&P Scientific). The beads were washed for 2 min by aspiration in 150 μL in the following wash solutions: loading buffer: 0.1 M glycolic acid, 80% ACN, 5% TFA; wash buffer 1:80% ACN, 1%TFA; wash buffer 2:10% ACN, 0.2%TFA, and they were finally eluted twice for 2 min in elution buffer: 1% NH_4_OH.

### High-pH Fractionation

For fractionation, we prepared a series of solutions with increasing concentrations of ACN in 0.1% triethylamine, following these steps: 3%, 6%, 9%, 12%, 15%, 18%, 21%, 24%, 27%, 30%, and 50%. We fractionated 50 μg of peptides on 5 mg of beads.

During each elution step, we resuspended the beads in the corresponding step elution buffer, transferred the suspension to the next well, and applied a vacuum to collect the eluate. For example, the peptides were initially loaded onto the beads in well A1. We then resuspended the beads in the first elution buffer (3% ACN), moved the beads to well A2, and applied vacuum to collect the first fraction in A2. Next, we resuspended the beads in the second elution buffer (6% ACN) and transferred them to well A3, applying vacuum to collect the second fraction in well A3, and so on for each subsequent step.

### Adipose Tissue-Conditioned Medium Digest Preparation

Human visceral and subcutaneous adipose tissue biopsies were cultured as 30 mg explants in 1 mL of serum-free DMEM/F-12 medium (Gibco 21041025) supplemented with penicillin/streptomycin in 6-well plates. The culture media were collected at 2, 24, and 48 h time points, filtered through a 0.22 μm filter, and stored at −20 °C in 500 μL aliquots.

The sample used in this study is a pool of four biological visceral fat replicates and one subcutaneous fat replicate. 100 μL of the sample was taken per analysis, reduced, and alkylated by adding TCEP to 10 mM and CAA to 20 mM for 30 min at 37 °C. Digestion was performed overnight by adding trypsin to 10 ng/μL and LysC to 5 ng/μL. The digest was desalted on 2 mg of the Oasis HLB Material.

### LC–MS Data Acquisition

Analysis of unmodified peptides was performed using a 300 μm column on a chromatography system operating at a flow rate of 5 μL/min. The Ultimate 3000 RSLC nano liquid chromatography system was coupled to either a Q Exactive HF-X or an Orbitrap Exploris mass spectrometer (both Thermo Fisher Scientific) via an EASY-Spray source. Electro-spray ionization was achieved by using Bruker PepSep emitters (part number: PSFSELJ20, 20 μm). Peptides were injected directly onto a self-packed CSH C18 column with 1.7 μm beads and dimensions of 300 μm × 30 cm. The peptides were separated using a 60 min stepped gradient that ranged from 0 to 45% buffer B over 70 min. The compositions of the buffers were as follows: buffer A consisted of 95/5%H_2_O/DMSO with 0.1% formic acid (FA), and buffer B consisted of 75/20/5% ACN/H_2_O/DMSO with 0.1% FA. The eluted peptides were analyzed in DIA mode. An initial MS1 scan was conducted at a resolution of 120,000, with an AGC target of 3 × 10^6^ and a maximum injection time of 200 ms, covering an *m*/*z* range of 410–1600. This was followed by 30 MS2 scans at a resolution of 30,000, covering the same *m*/*z* range with variable windows predicted using encyclopeDIA.^32^ The AGC target for the MS2 scans was 3 × 10^6^, the maximum IT set to auto, and the normalized collision energy was set to 27.

Analysis of phosphopeptides was performed using a 150 μm column on a chromatography system operating at a flow rate of 1.25 μL/min. The Ultimate 3000 RSLC nano liquid chromatography system was coupled to an Orbitrap Exploris 240 mass spectrometer via an EASY-Spray source. Electro-spray ionization was achieved by using Bruker PepSep emitters (part number: PSFSELJ10, 10 μm). Peptides were injected directly onto a self-packed CSH C18 column with 1.7 μm beads and dimensions of 150 μm × 30 cm. The peptides were separated using a 60 min stepped gradient that ranged from 0 to 45% buffer B over 70 min. The compositions of the buffers were as follows: buffer A consisted of 95/5%H_2_O/DMSO with 0.1% formic acid (FA), and buffer B consisted of 75/20/5% ACN/H_2_O/DMSO with 0.1% FA. The eluted peptides were analyzed in DIA mode. An initial MS1 scan was conducted at a resolution of 120,000, with an AGC target of 3 × 10^6^ and a maximum injection time (IT) of 200 ms, covering an *m*/*z* range of 410–1600. This was followed by 25 MS2 scans at a resolution of 30,000, covering the same *m*/*z* range with variable windows predicted for phosphopeptides using encyclopeDIA.^32^ The AGC target for the MS2 scans was 3 × 10^6^, the maximum IT was set to auto, and the normalized collision energy was set to 27.

### Data Analysis

Data were processed using the Spectronaut software platform (Biognosys, 19.9.250324.62635). Analysis was carried out in direct DIA mode using the stringent settings described previously by Baker et al., PSM, Peptide, and Protein group identification FDR = 0.01.^33^ Searches were carried out against the one entry per gene UniProt *H. sapiens* (retrieved 2024 09 30), *E. coli* (retrieved 2024 09 24), and *S. cerevisiae* (retrieved 2024 02 05) databases. The quantification method was configured as follows: MS2-based quantification with a proteotypicity filter set to “Only Proteotypic” and the label-free quantification (LFQ) method set to MaxLFQ. No value imputation was employed. For phospho-site analysis, default settings were used for PTM analysis, with the “Multiplicity” option enabled in PTM Workflow, to enable phosphopeptide precursor collapse to site level with consolidation set to sum.

## Results and Discussion

### Introducing Oasis HLB Self-Packed Plates

One of the most common approaches in proteomics sample preparation (and an integral part of phosphoproteomics) is sample cleanup using SPE, and we aimed to perform this in a 96-well plate format. While commercially available plates are cost-effective when processing a full set of 96 samples (typically $300–$400 per plate, or $3–$4 per sample), the situation becomes problematic when only part of a plate is required. We frequently encountered sample sets that were smaller than 96 samples—or larger but not divisible by 96. For example, processing 120 samples would require the use of one full plate and only 24 wells of a second plate. Such partial plate use either forces the disposal of a nearly unused plate, significantly increasing the per-sample cost, or pushes the workflow back toward individual tubes.

In principle, automation could solve this by having a liquid handler reformat samples before and after desalting. However, the 96-channel devices described here cannot perform such operations, requiring users to manually reformat plates, which substantially increases both labor and the risk of error. A far more practical solution is to produce low-cost plates by packing SPE material into filter plates, making it feasible to discard partially used plates without major cost implications.

Oasis HLB material has become popular in proteomics, with most research groups eventually transitioning to it from earlier C18 cartridges. The main challenge with the C18 material is that it is not water-wettable. When analytes are bound to the C18 sorbent, the aqueous solvent is removed (through positive or negative pressure or centrifugal force), and the C18 phase dries out, leading to irreproducible recoveries.^34^ Oasis HLB is a copolymer of two monomers—lipophilic divinylbenzene (DVB) and hydrophilic *N*-vinylpyrrolidone (NVP)—each contributing a distinct interaction mode. The DVB component provides strong hydrophobic retention, while NVP introduces polar and hydrogen-bonding interactions that enhance the retention of more hydrophilic analytes. Together, this balanced mixed-mode chemistry enables purification of a broad range of diverse compounds^35^ Additionally, this composition allows it to be wettable in both water and organic solvents. As a result, when it is left in an aqueous environment, it does not dry out, leading to more reproducible recoveries.

To create an affordable Oasis HLB plate (at the time of publication, the final price is around 25$) that operates in a 96-well format, we packed 30 μm bulk Oasis HLB material into an OROF1100 filter plate. This plate features a polypropylene 10 μm filter, and the wells are tapered for optimal performance.

Supporting Information Video S1 illustrates the plate manufacturing process, and Figure 1A displays a plate packed with 30 mg of the material (600 μL of 50 g/L bead slurry).

**Figure 1:**
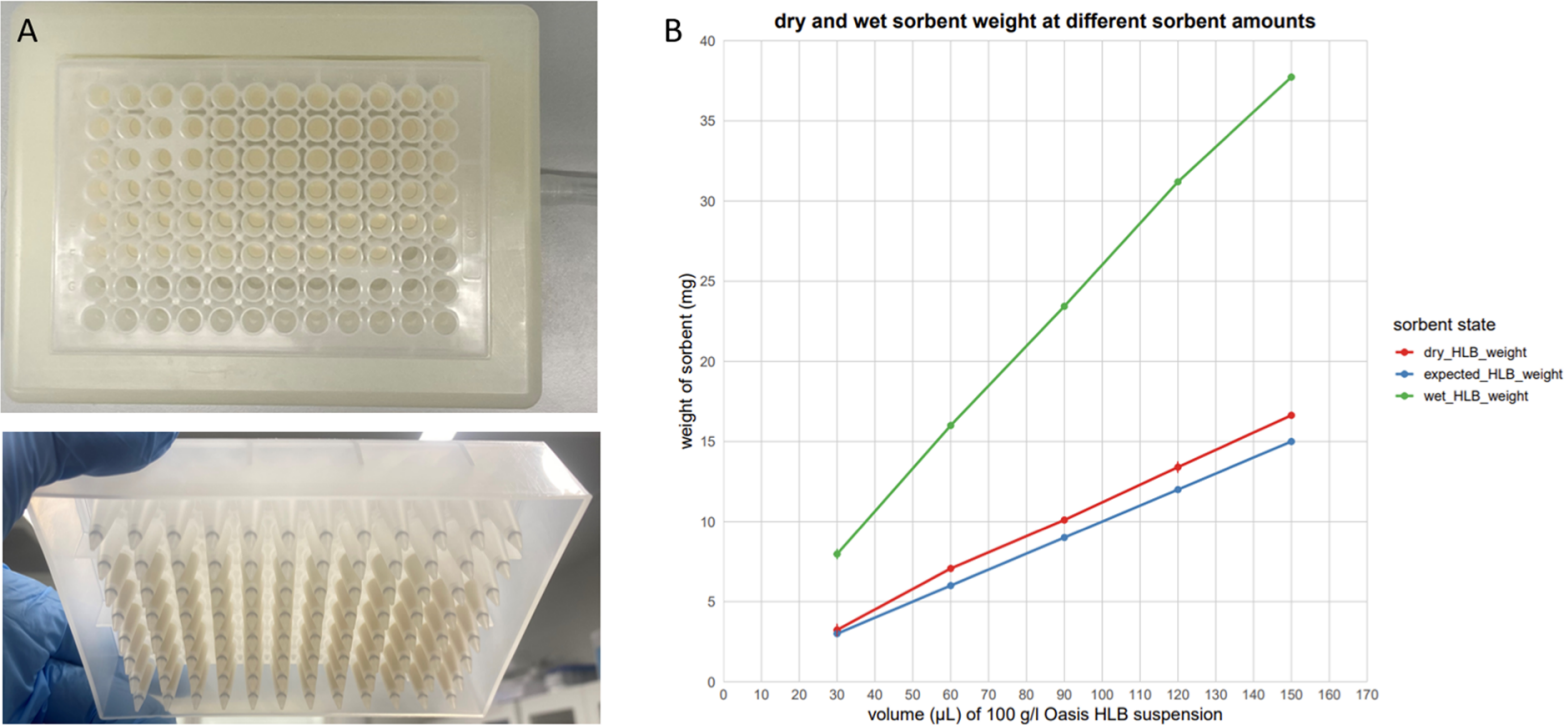
Self-packed Oasis HLB plate. (A) An Orochem OF1100 plate packed with 30 mg of material into A1-F10 positions, designed for processing 70 samples; photo taken by the authors. (B) Investigation of liquid retention by a given amount of Oasis HLB material. The material was weighed while wet, with aqueous solution, and after drying.

### Each mg of Oasis HLB Material Retains 1.3 μL of Aqueous Solution

One critical aspect of the material we characterized is the amount of aqueous liquid retained by it. In phosphoproteomics enrichment using Zr-IMAC beads, for example, peptides need to be desalted after digestion and then loaded onto the beads in 80% ACN. Using lower concentrations results in suboptimal recoveries.^36^ To achieve the precise ACN concentration post-desalting, one option is to evaporate the eluate and then resuspend it in the solvent of desired composition, but this introduces a lengthy additional step. Instead, by determining the exact amount of liquid retained by the beads, we could simply add ACN directly to the beads to reach the final concentration of 80% and perform phosphoenrichment directly on the desalting eluate.

To investigate the degree of liquid retention by the Oasis HLB material, we weighed 15 empty filter tips and loaded them in triplicate with 30, 60, 90, 120, and 150 μL of a 100 g/L suspension material (which corresponds to the expected material weights of 3, 6, 9, 12, and 15 mg). After loading, we equilibrated the material in the tips with 0.1% TFA and subjected the tips to centrifugation for 10 min at 3000*g*. Despite extensive centrifugation, the sorbent remained wet, as anticipated. We weighed each tip again with a wet sorbent. We then re-equilibrated the sorbent with ACN, dried the contents using sepeed-vac and weighed the tip one more time with dry sorbent. Knowing the weight of the tip allowed us to calculate the masses of wet and dry sorbents as shown in Table S1 and Figure 1B. The weight of the dry sorbent is close to expected; however, the weight of the wet sorbent is approximately 2.3 times higher. This indicates that every 1 mg of sorbent retains about 1.3 mg, or 1.3 μL, of 0.1% TFA.

Hence, if we loaded 300 μg of peptides onto 30 mg of the sorbent (see below), the sorbent would retain 39 μL of 0.1% TFA. Therefore, to elute these peptides using 600 μL of 80% ACN, we would need to add 480 μL of ACN and 81 μL of 0.1% TFA (a total of 120 μL of aqueous solution is required; 39 μL is retained by 30 mg of Oasis HLB material.

### Desalting Should Be Performed at a 1:100 Peptide-to-Sorbent Ratio

Next, we tested the loading capacity of the HLB material for the peptide analysis. According to the manufacturer, the recommended loading capacity is between 1 and 3%, though it can be as high as 10%.^37^ We used 1 mg of sorbent and conducted duplicate desalting for 100, 50, 33, 25, 20, 16.7, 12.5, 10, 8, 6.7, 5.7, and 5 μg of peptides, resulting in sorbent-to-peptide ratios (w/w) of 10, 20, 30, 40, 50, 60, 100, 125, 150, 175, and 200 to 1, respectively. Notably, the amount of peptide material was calculated based on the BCA assay at the protein level before protein precipitation and digestion, which might not be reflective of the final amount of material at the peptide level. We compared the signal of 1 μg of the desalted sample (at a 1:100 sample-to-medium ratio) and 1 μg of a commercial HeLa standard from Thermo, and both demonstrated nearly identical signal levels (data not shown).

Both the flow-through and desalted samples were collected, dried under vacuum, and resuspended in 0.1% TFA. We then analyzed 1 μg of material, assuming complete recovery during the cleanup procedure for desalted samples or a complete lack of retention for the flow-through samples. To our surprise, all the desalted samples had highly similar proteomic profiles, and they did not show any clustering on the PCA plot (Figure 2A).

**Figure 2:**
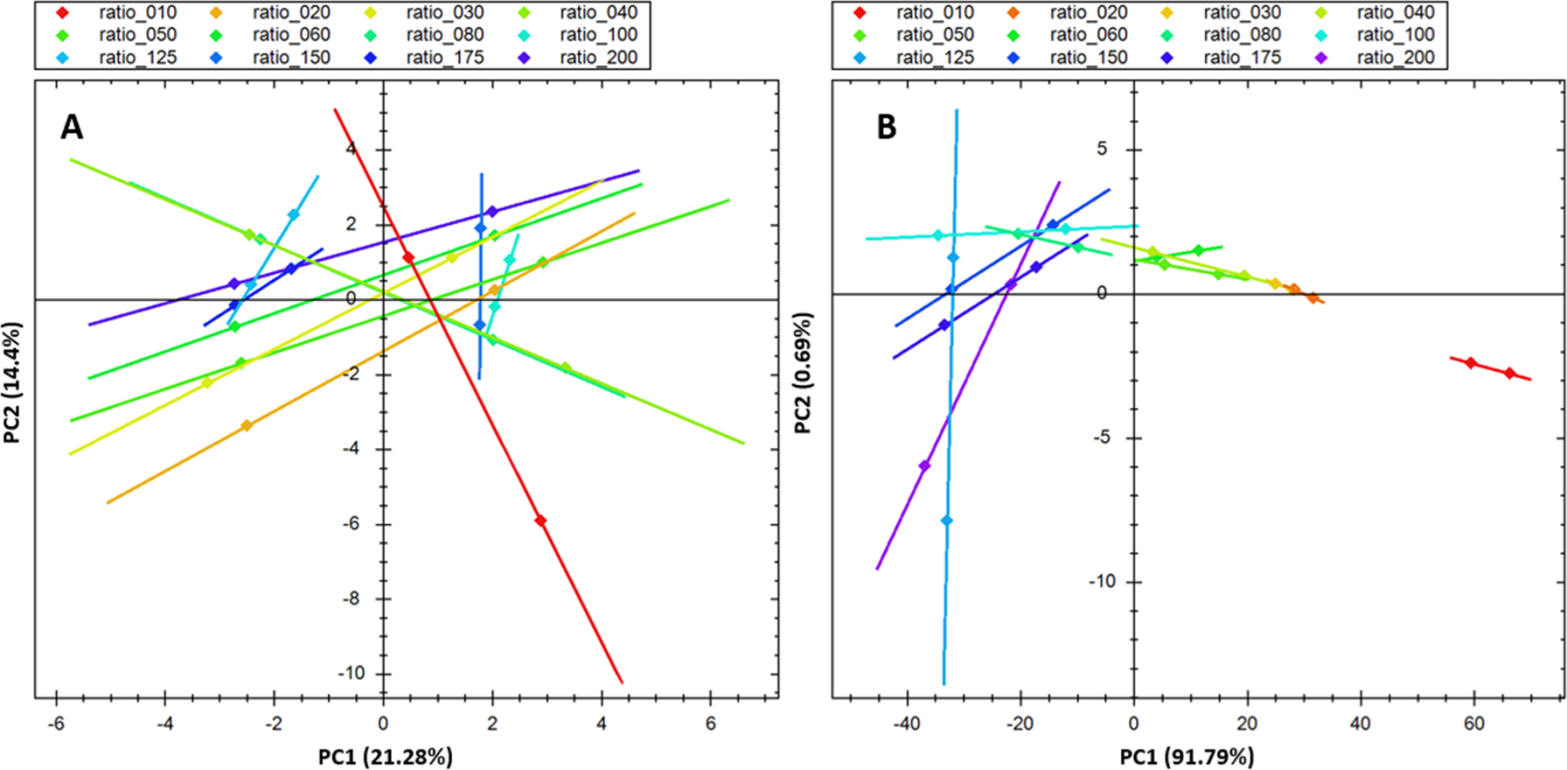
Effect of the peptide-to-bead ratio on desalting. (A) As measured on the desalted THP1 sample. (B) As measured on the flow-through.

We then analyzed the flow-through samples and observed significantly higher levels of flow-through in 1:10 samples, compared to other samples, with nonretained peptides mostly observed in the hydrophilic range. This indicates, as expected, that when the material is overloaded, the peptides with weaker interaction with the material lose their retention first. As the ratio of the HLB material to peptide increased, we observed that the signal of flow-through stabilized at 1:100, with higher ratios (1:125, 150, 1:175, and 1:200) being indistinguishable from 1:100 (Figure 2B).

### Oasis HLB Material Is Not Effective in Removing Lipid Contamination

C18 can be used for cleaning lipid contamination from peptide samples. As an example, the increasingly popular evoSep platform utilizes the principle of “partial elution”, whereby peptides are eluted from a disposable trap column using a gradient of up to 35% of ACN, while lipid and polymer contaminants remain bound to the trap column.^8^ This method results in a significantly extended lifespan for the analytical column with no increase in backpressure even after thousands of runs.

One of our projects involved analyzing adipose tissue-conditioned cell culture media, where we observed significant lipid contamination. To address this issue, we similarly tried to determine the concentration of ACN at which peptides elute from the Oasis HLB material, while lipids remain bound to it. To achieve this, we tested in duplicate the elution from 2 mg of the material with ACN concentrations of 15%, 20%, 25%, 30%, 35%, 40%, 45%, 50%, 55%, 60%, 65%, and 70% ACN using 100 μL of conditioned media pooled from 18 samples representing visceral and subcutaneous fat. Additionally, we analyzed a 20 μg THP-1 digest under the same conditions. Based on the number of identifications and sample clustering, we found that full peptide elution could be achieved at 35% ACN (see Figure 3A,B). However, the lipid signal at the 35% concentration was as high as it was at 70% ACN (see Figure S1). Lower lipid retention is expected because the mixed-mode chemistry of Oasis HLB provides weaker binding to hydrophobic species than conventional C18 sorbents.^38^ As a result, lipids elute at the same ACN concentration as peptides, making peptide–lipid separation by ACN content modulation on the Oasis HLB material impossible.

**Figure 3:**
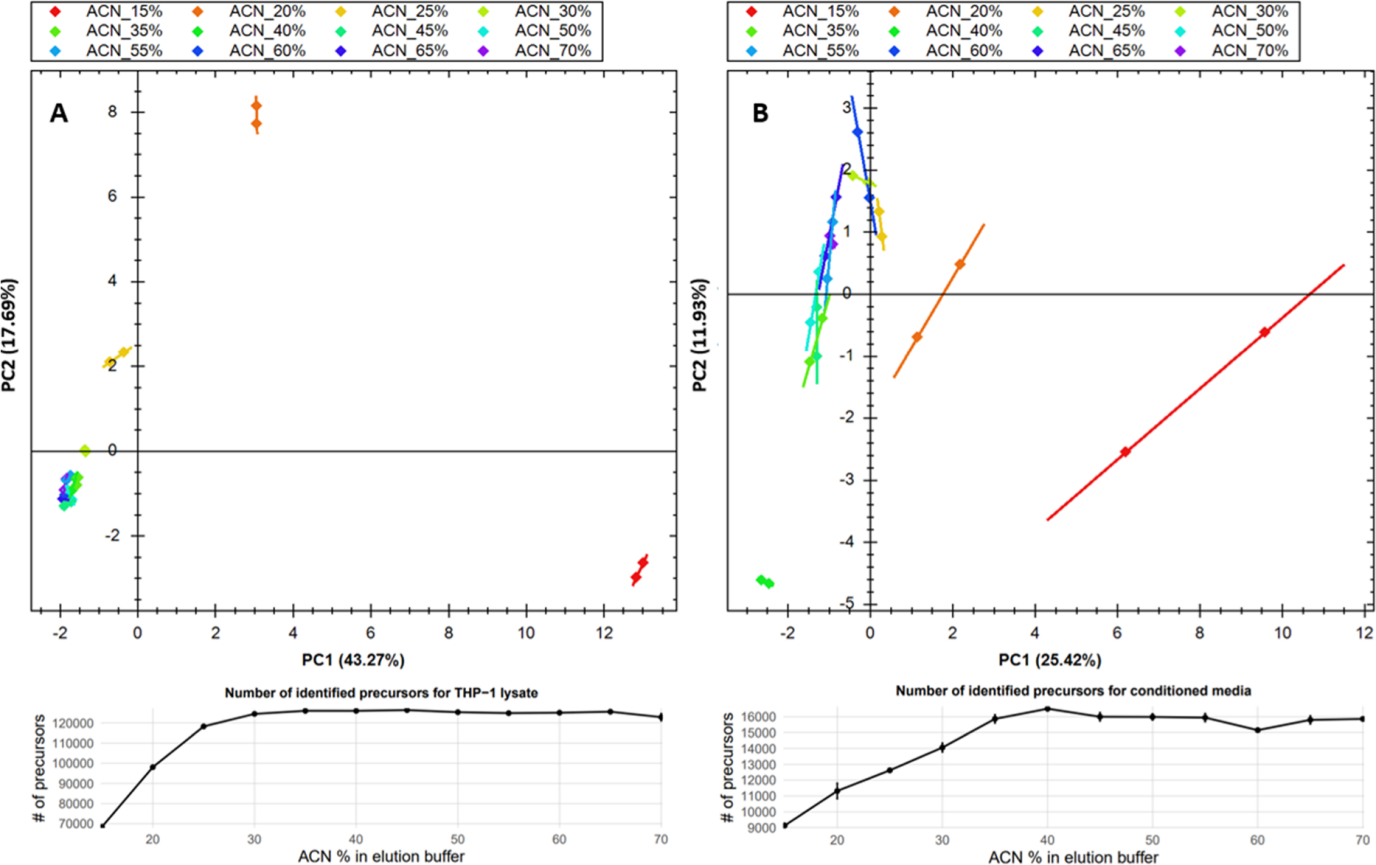
Optimization of sample elution from Oasis HLB beads. (A) For THP-1 digest. (B) For adipocyte conditioned media.

### Oasis HLB Material Is Inferior to C18 for High-pH Fractionation

In bottom-up proteomics, peptides are typically separated by low-pH RP chromatography before electrospray ionization and mass spectrometric analysis. To improve proteome coverage, peptides can be prefractionated using an orthogonal separation method, with each fraction subsequently analyzed in a separate LC–MS run. These orthogonal separation methods are typically evaluated based on the number of fractions they can generate with minimum identification overlap between fractions. As an example, RP chromatography at high pH is the most widely adopted method because it is highly orthogonal to online low pH chromatography and because it is possible to generate multiple >40 fractions with 70–80% of peptides being detected in only a single fraction.^39^

However, C18 is not water-wettable, requiring solvents to be passed through it at high pressure, either by using an LC system or centrifugation.^40^ To simplify fractionation, the Olsen group, in collaboration with Resynbio, used magnetic HILIC beads to fractionate the peptides into five fractions. The premise of this fractionation method is that the beads can be resuspended in each elution solution, making the fractionation very quick: beads are resuspended by aspiration, eluting a portion of peptides; the beads are bound to the magnet; the eluate is transferred to a new tube; the beads are resuspended in the next elution solution; etc. The authors indicated that the overlap between fractions was significantly higher than with high pH RP, with around 50% of the peptides unique to a single fraction (personal communication).

Given that the Oasis HLB material is also wettable in any combination of organic and aqueous solvents, we reasoned that it could similarly serve for easy high-pH fractionation by repeatedly resuspending the beads with bound peptides in a series of solutions with increasing ACN concentration. With every step, we moved the beads to a new well and collected the fraction to the plate underneath by application of vacuum (see the Methods section for details). The fractionation procedure was very easy to execute, and using a multichannel pipette, it could be applied to 8 samples at a time. We observed that the peptides eluted much earlier than in conventional C18, with a high proportion of peptides already eluting at 3% ACN, indicating that peptide positive charge is what drives a lot of interactions with Oasis HLB (Figure 4A; the experiment was conducted in a single replicate). Unfortunately, we observed that the fractions demonstrated too much overlap, with only 30% of peptides unique to a single fraction (Figure 4B); hence, we concluded that while high-pH fractionation on the Oasis HLB material is possible, it is suboptimal compared to conventional C18 and the HILIC procedure mentioned above. The explanation likely stems, once again, from the dual retention mechanism of Oasis HLB, as opposed to the purely hydrophobic character of C18. In standard C18, the orthogonality between low- and high-pH reversed-phase separations arises from pH-driven changes in peptide charge: e.g., when basic residues become neutral at high pH, peptide hydrophobicity increases, requiring higher ACN to elute them.^41^ A similar effect is expected when peptides interact with the hydrophobic DVB component of the Oasis. In fact, polystyrene–divinylbenzene (sDVB) has been reported to be an excellent stationary phase for high-pH reversed-phase fractionation.^42^ However, the Oasis HLB also contains a hydrophilic NVP moiety, and peptide interactions with this component are largely pH-independent. While this enhances peptide recovery under diverse loading conditions, it also diminishes the retention differences between high- and low-pH modes, ultimately reducing separation orthogonality.

**Figure 4:**
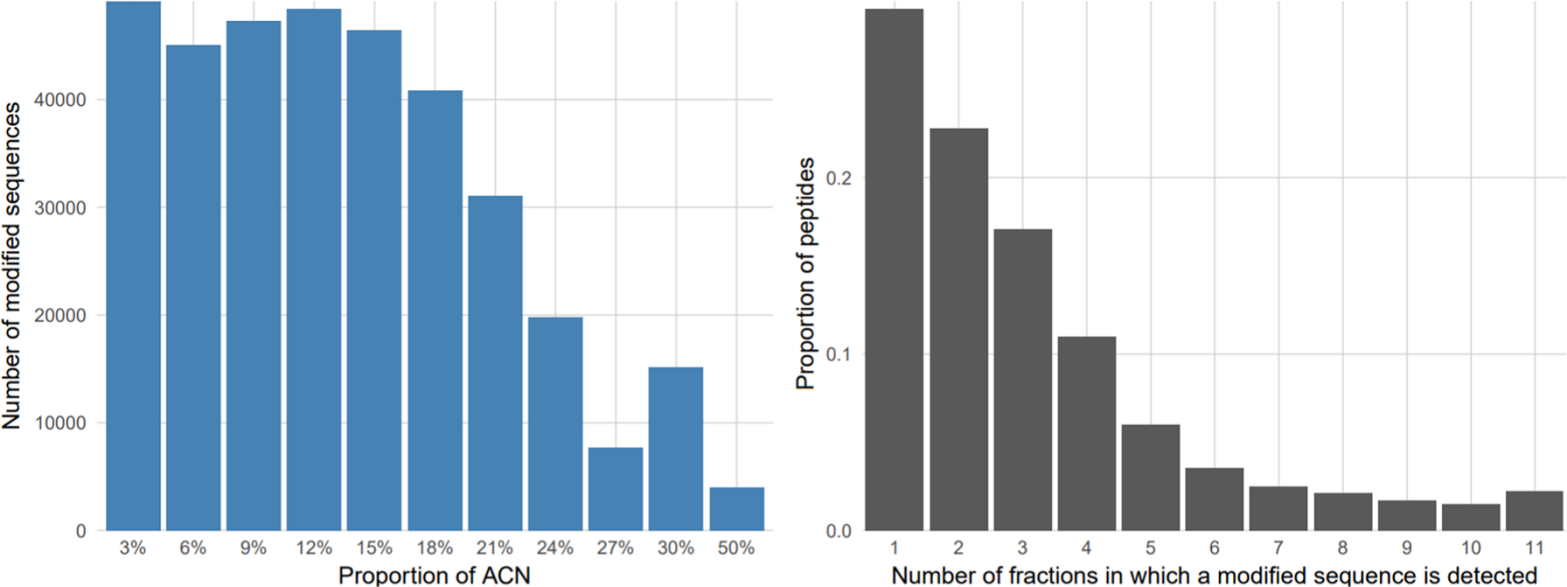
Evaluation of high pH peptide fractionation on the Oasis HLB material. (A) Number of identified peptides eluted at a certain proportion of ACN. (B) Proportions of peptides observed in a certain number of fractions.

### Pipeline for Phosphoproteomics Using 96-Well Channel Devices

The entire pipeline for phosphoproteomics analysis is illustrated in Figure 5. Platemaster P220 is utilized throughout the workflow, but every step requires different accessories and consumables that we have carefully selected through several years of optimization.

**Figure 5:**
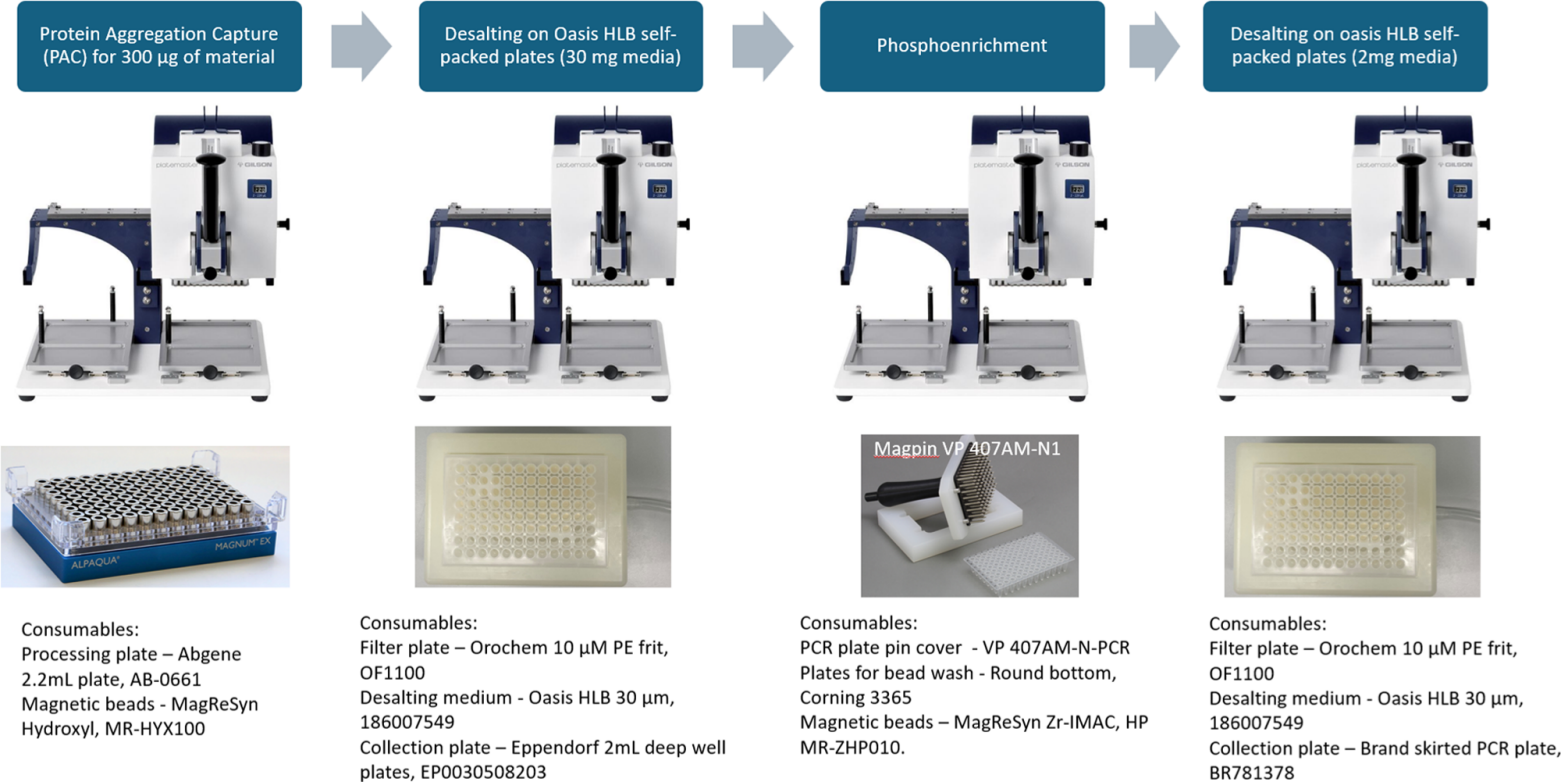
Devices and consumables used at every step of phosphoproteomics processing.

One important aspect of the workflow is the desalting step after postphosphoenrichment. Initially, we tried to avoid this desalting step, since in principle, the only reagents present in the phosphoenrichment eluate should be peptides and ammonium formate (peptides are eluted in 1% ammonia, which is then neutralized with formic acid), and ammonium formate is not retained by RP columns. Hence, we expected to simply speed-vac the eluate from Zr-IMAC beads, resuspend it in the desired volume of 0.1% TFA, and load it directly onto the column. However, we found that chromatography quickly deteriorates when injecting nondesalted samples, especially since we operate in direct injection mode (see Figure S2). Therefore, we always perform a desalting step after phosphoenrichment.

### Digestion in PAC Can Be Accomplished by Short Pipetting

We describe the PAC procedure below, and it is remarkably simple to execute in a 96-well format, even for inexperienced operators. However, we found one step to be particularly challenging: digestion. Initially, we assumed that to digest the proteins off the beads, we needed to keep the beads in suspension. For overnight digestion, this required shaking on an orbital shaker. However, finding the correct RPM for shaking proved to be quite difficult. Shaking at low RPM meant that the beads were not resuspended, while shaking at too high RPM caused the solution to splash in the well, leading to potential cross-contamination and sample losses due to drying. The two shaking set-ups that consistently worked are shown in Table 1.

**Table 1:** Recommended Setups for Bead Shaking That Keep the Beads in Suspension without Excessive Splashing

	small-scale digestion	large-scale digestion
amount of material	50 μg	300 μg
digestion volume	50 μL	300 μL
plates	BR781377 plates with AM-96-PCR-RD mats	AB-0661 with 30127960 mats
RPM for the orbital shaker	1650	1200

Since finding the proper RPM for bead shaking proved to be quite challenging, we decided to test whether simply pipetting the beads up and down would be sufficient. Interestingly, the Batth paper,^12^ which demonstrated that the PAC mechanism is driven by protein aggregation, also noted that the digestion efficiency in the PAC workflow is very high. This results in significantly lower miscleavage rates and effective digestion, even when using 10–20 times less trypsin compared to an in-solution digest.^12^ We hypothesized that the PAC digestion is so effective since the exposed protein pellet area is maximized in PAC, due to proteins covering the beads in a thin film, and hence, a very quick step of repeated pipetting or shaking for a limited time could result in efficient digestion.

We digested 50 μg of the THP-1 lysate on beads in triplicate, testing both shaking (for durations of 0.5, 1, 2.5, 5, 10, 25, 50 min, and overnight) and pipetting (for durations of 0.5, 1, 2.5, 5, and 10 min). One triplicate was left unshaken and unpipetted (0 min). After being shaken or pipetted, beads were allowed to settle and stayed at the bottom of the well during overnight digestion.

Importantly, after the final PAC wash and before the addition of the protease, the plate was briefly centrifuged to ensure that the beads moved to the bottom of the well. This step ensured that the beads were not resuspended when adding the protease solution to the 0 min time point samples, and we took extra care to ensure that the beads were not resuspended at any point for these samples.

The results are displayed in Figure 6A, with several notable observations. First, the samples that were shaken overnight were separated from the other sample preparation groups in the first principal component (PC1). In addition, the within-group variance was highest in the overnight digestion, likely due to the suspension splashing against the sides of the container during digestion. This indicates that overnight shaking is excessive, and when using a heater–shaker for digestion, the shaking duration should be limited to no more than 2 min. Notably, samples with pipetting show less within-group variability than samples with shaking. Interestingly, even just 1 min of shaking or pipetting is sufficient for optimal digestion, as the 1 min treatments cluster closely with their corresponding groups. Finally, we were surprised to observe that even without resuspending the beads (0 min), we still achieved a significant recovery, further validating that it is not necessary to keep the beads in suspension during digestion. Digestion efficiency was assessed by the proportion of missed-cleavage peptides (Supporting Information Figure S3A) and their contribution to the total signal (Supporting Information Figure S3B). Across all samples, the fraction of miscleaved peptides was similar, with overnight shaking causing a modest reduction in the number of missed cleavages.

Efficient digestion with just a few minutes of shaking or pipetting could be explained in two different ways. First, Brownridge et al. demonstrated that many peptides exhibit extremely rapid digestion kinetics; hence, introducing several rapid cleavages to the protein might be sufficient to remove it from beads, with more challenging cleavages occurring later overnight in solution.^43^ Hence, hypothetically, the proteins might be stripped off the beads after just 2 min of pipetting. Second, it is possible that the beads need to be fully wetted with the protease solution, with digestion primarily occurring while the proteins remain bound to the beads. These two possibilities can be distinguished experimentally: if proteins are released into the solution during the initial pipetting, the supernatant could be collected and left overnight for further digestion without a loss of signal. Conversely, if proteins remain on the beads after the initial pipetting, leaving the beads in solution overnight should improve recovery.

To differentiate between these two scenarios, we performed PAC digestion on 30 μg of the THP1 lysate in duplicates. Digestion was carried out by pipetting the beads up and down for 0.5, 1, 2.5, 5, 7.5, or 10 min, followed by a 30 s period to allow the beads to settle at the bottom of the well. For half of the samples, digestion continued, with the beads remaining in the protease solution. For the other half, the beads were removed using a magnet, allowing digestion to proceed, with only the proteins released into the supernatant. In both cases (with beads left in solution and with beads taken out), digestion was allowed to continue overnight.

The PCA plot showed clustering of samples based on whether beads were retained or removed during digestion (Figure 6B). Bead presence had no impact on digestion efficiency, as measured by missed cleavages (Supporting Information Figure S4). In contrast, we observed marked differences in signal levels between samples with beads left in and those from which beads were removed, judged by precursor intensity distributions (Supporting Information Figure S5A). To assess the effect of bead handling on individual precursor signal recovery, we calculated, for each precursor, the ratio of its intensity with beads retained versus beads removed after pipetting for a given amount of time and plotted the distribution as boxplots (Supporting Information Figure S5B). Recoveries increased from roughly 32% after 0.5 min of pipetting to about 79% at 10 min (Supporting Information Figure S4B). These results support the second hypothesis, indicating that a significant fraction of proteins remains bound to the beads even after 10 min of pipetting in the digestion buffer. Consequently, efficient digestion with brief, e.g., 2 min, pipetting is achieved by saturating the beads with protease solution, rather than by fully releasing the protein precipitate from the beads.

**Figure 6:**
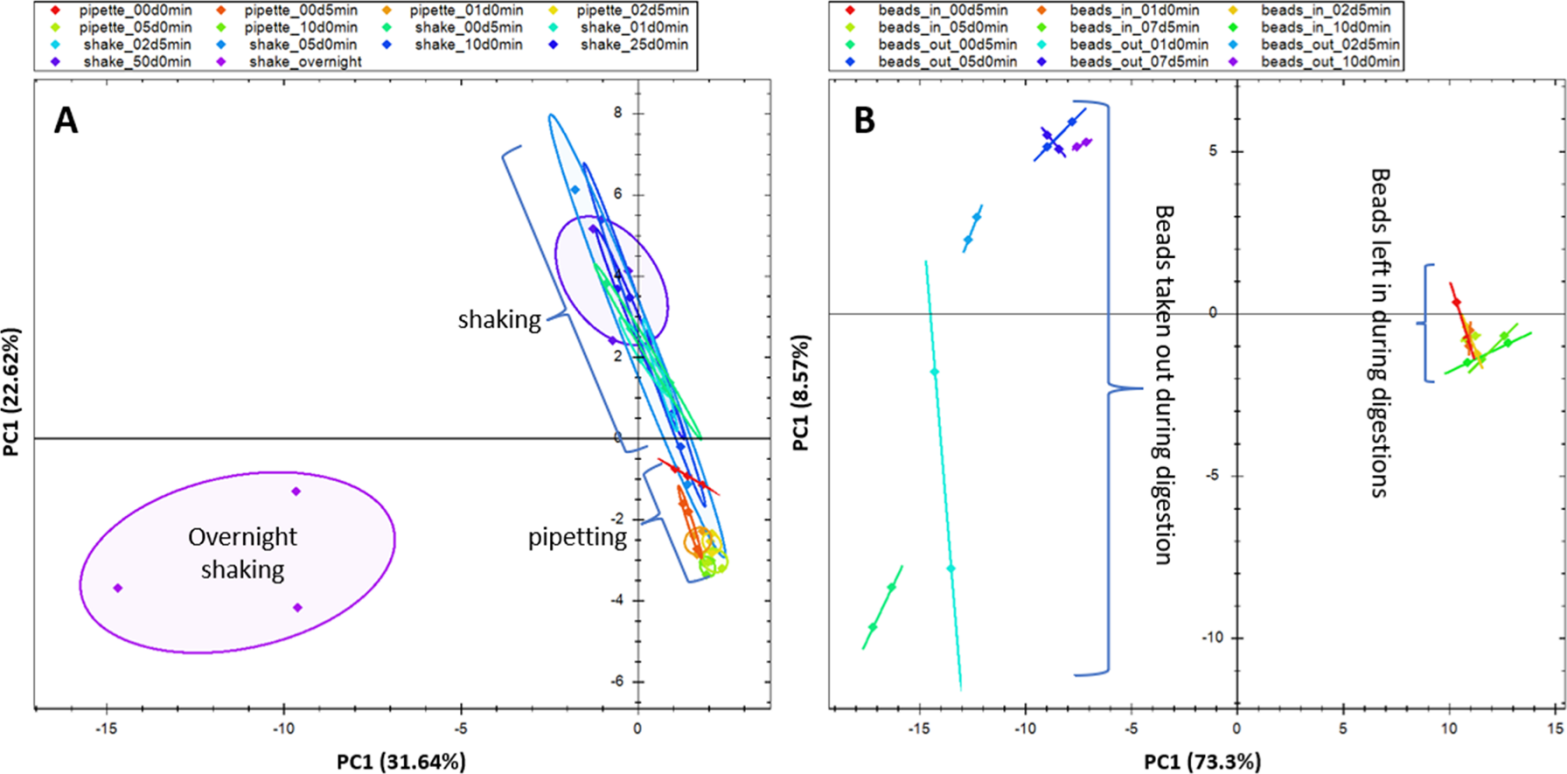
Digestion using pipetting or shaking to keep beads in suspension. (A) PAC digestion samples shaken overnight demonstrate the most variability and cluster away from pipetted and shaken samples (B). PAC digestion achieved by pipetting beads in protease solution for varying durations, with beads either removed or retained after settling.

In the experiments described below, we did not shake the beads after protein precipitation; instead, we aspirated the solution up and down for 2 min and then allowed the beads to settle during the overnight digestion.

### Benchmarking PAC and Phosphoproteomics with a Three-Species Mix

The PAC procedure is best understood by watching Video S2 and is also illustrated in Figure 7A. Platemaster P220 has four positions designated for waste basin, tip wash basin, PAC plate with resynbio hydroxyl beads covered with the protein precipitate, and wash solution (80% ethanol). In principle, the consumables can be arranged in any order, but we found this arrangement the most natural. The wash cycle consists of five steps: 1. Transfer the wash solution to PAC plate 2. Wash the PAC beads by aspiration. 3. Place the plate on a magnet. 4. Aspirate and discard the wash solution. 5. Wash the tips.

**Figure 7:**
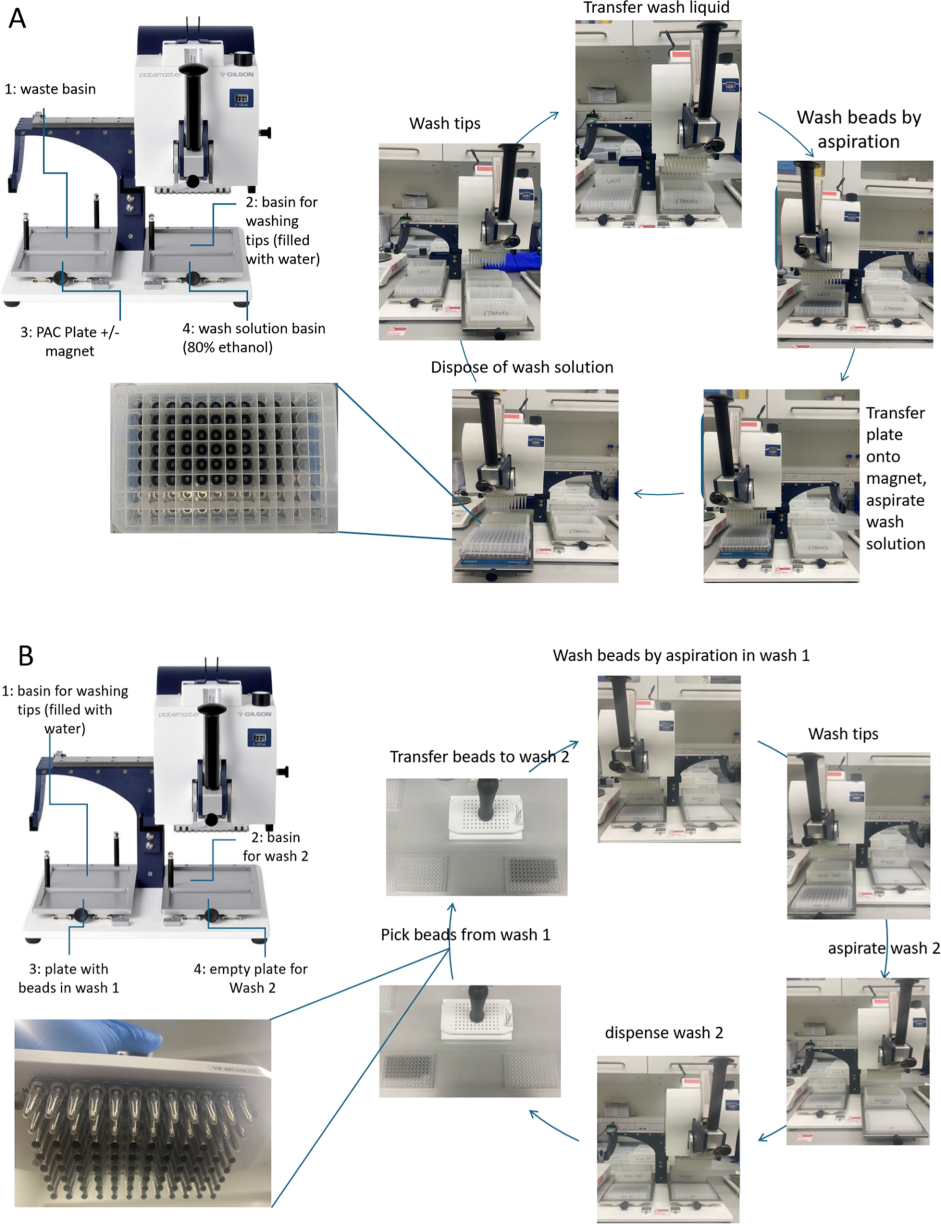
Using magnetic devices for proteomics sample preparation. (A). PAC cycle explanation. (B). Phosphoenrichment cycle explanation.

After completing the washes, we perform digestion by aspirating the beads up and down in a solution containing 20 ng/μL trypsin and 10 ng/μL LysC for 2 min (a final protein-to-trypsin ratio of 50:1 and a protein-to-LysC ratio of 100:1). Notably, the only step in the PAC procedure that cannot be performed with the 96-channel pipettor is the addition of proteases since filling a 96-channel reservoir with proteases is prohibitively expensive. Therefore, we add the proteases using 12-channel pipettes and then proceed to pipetting the beads up and down for 2 min and allow the beads to settle to the bottom of the wells for overnight digestion.

The phosphoenrichment process begins with 300 μg of peptides eluted in 600 μL of 80% ACN. To this solution, we add glycolic acid to 0.1 M and TFA to a 5% final concentration in a single step. The phosphoenrichment procedure is best understood by watching Video S3 and is also illustrated in Figure 7B, and it consists of five washing steps (see the Methods section). Platemaster P220 has four positions designated for the tip wash basin filled with water, the basin containing wash #2, the plate containing the Zr-IMAC HP beads in wash #1, and an empty plate for wash #2. The wash cycle (illustrated for transferring beads from wash #1 to wash #2) is as follows: 1. Wash the beads in wash #1 for 2 min by pipetting. 2. Wash the tips. 3; Aspirate wash #2. 4. Fill plate #2. 5. Remove the beads from plate #1 with a MagPin device. 6. Release the beads to plate #2.

To benchmark our setup, we prepared a set of three proteome mixes consisting of proteins from *E. coli*, *S. cerevisiae*, and *H. sapiens*. In these mixtures, the amount of *E. coli* was kept constant, while the proportions of the *S. cerevisiae* and *H. sapiens* proteomes were varied (see Figure 8A). Each mixture was prepared in 10 replicates, resulting in a total of 70 samples. Once the samples were digested, a portion of each sample was mixed to create a pooled sample, which was subsequently injected after every mix replicate to assess the contribution of LC–MS to quantitation variability. The results are shown in Figure 8B,C, where the proteome and phosphoproteome samples demonstrate the expected clustering. Figure 8D presents boxplots comparing observed versus expected ratios for the PAC processing, highlighting that the experiment accurately recovered the expected ratios.

**Figure 8:**
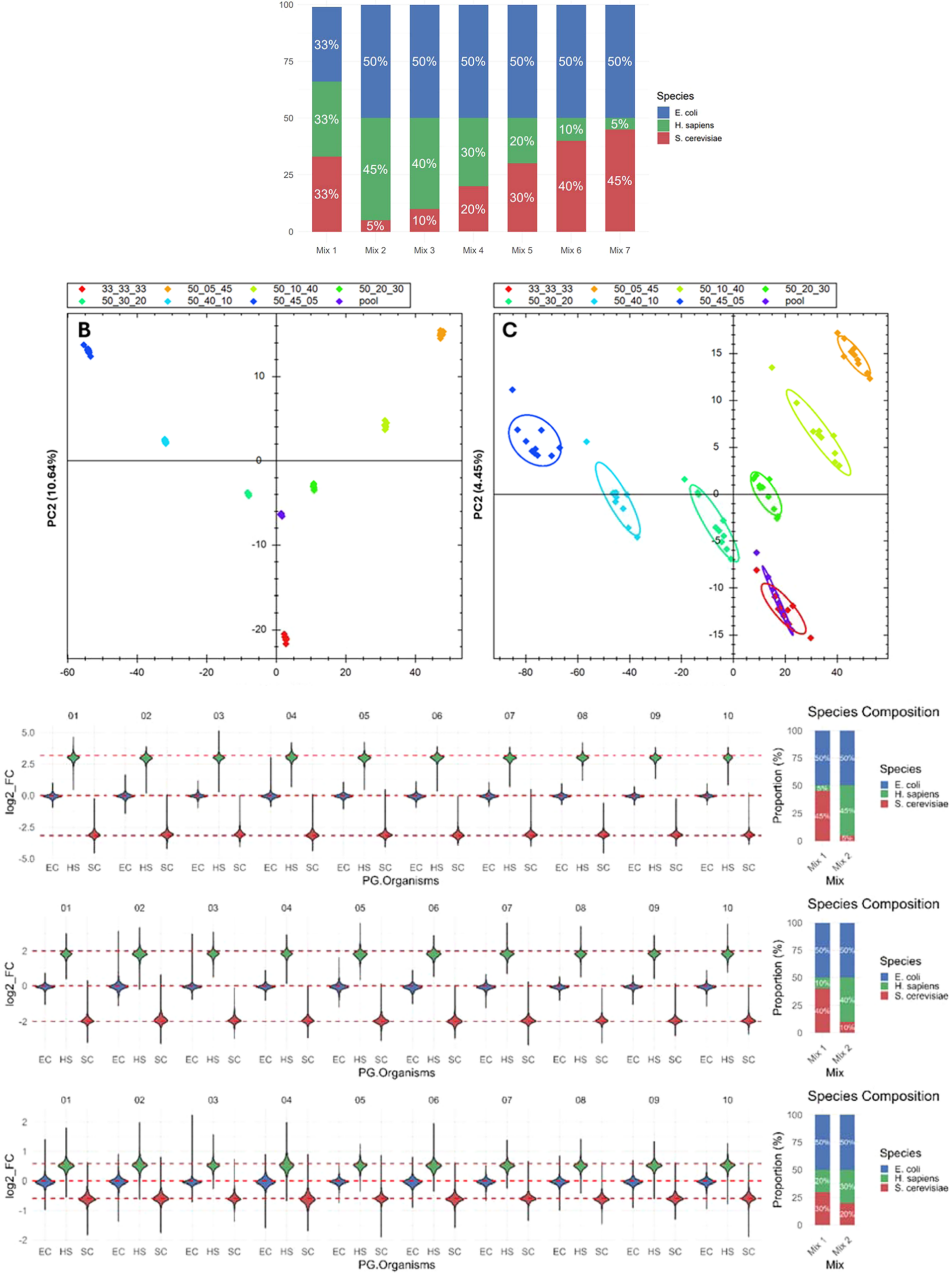
Evaluation of the phosphoproteomics pipeline using a three-species mixed experiment. (A) Mixing schema. (B) PCA plot illustrating total protein level quantification. (C). PCA plot for phosphoproteomics analysis. (D) Visualization of recovered ratios by comparing selected mixtures. Note that the mixes were compared within their preparation replicates. For instance, the 50:45:5 mix replicate 1 was compared to the 50:5:45 mix replicate 1.

Table 2 provides a more detailed summary of the results, including the number of quantified proteins and sites with complete profiles, missed-cleavage rates, and, importantly, coefficients of variation (CVs) for quantitation. The total CVs capture the combined variability from both sample preparation and LC–MS data acquisition. By including repeated acquisitions of a pooled sample, the variability attributable to LC–MS acquisition can be estimated, allowing isolation of variability introduced by sample processing. The CVs observed for the mixed proteome samples are higher than those for the pooled sample, indicating that the described sample processing workflow contributes around ∼1–2% to protein-level CVs and ∼2–3% to phosphosite-level CVs. Finally, Table S2 reports the root mean square error (RMSE) of the expected protein ratio measurements. The normalized RMSE (RMSE divided by the expected ratio) ranges from 0.1 to 0.2, reflecting good agreement between the observed and the theoretical ratios.

**Table 2:** More Detailed Summary of the Results, Including the Number of Quantified Proteins and Sites with Complete Profiles, Missed-Cleavage Rates, and, Importantly, Coefficients of Variation (CVs) for Quantitation

mix (EC_ SC_HS)	missed cleavage rate	protein CV	number of proteins with full profiles	phospho-site CV	number of quantified phospho-sites
33_33_33	15.7 ± 0.62	7.7	8055	19.2	4559
50_05_45	15.03 ± 0.67	7.76	6906	17.9	4298
50_10_40	15.35 ± 0.7	7.49	7319	18	4333
50_20_30	15.62 ± 0.69	7.46	7630	20.2	4113
50_30_20	15.83 ± 0.57	6.99	7490	21.4	3675
50_40_10	15.83 ± 0.63	6.72	6738	20.9	2860
50_45_05	15.78 ± 0.65	7.53	5708	26.5	2103
pool	15.44 ± 0.86	6.04	7787	17.7	3686

## Discussion

Here we describe a method for preparing samples for proteomics and phosphoproteomics using 96-channel devices. This manuscript summarizes more than three years of method development and experience we gained from using this setup in numerous projects run at the MRC Laboratory of Medical Sciences proteomics facility.

An important aspect of our optimization is showing that the beads do not need to stay in solution during the digestion process. Instead, they can settle after just 2 min of aspiration or shaking. This significantly simplifies the automation of the PAC procedure by eliminating the need for a heater–shaker. Notably, in the original paper that described PAC automation on the Opentrons OT-2, Liu et al. assumed that effective digestion requires shaking the plate.^24^ As a result, they were unable to complete the protocol entirely on the OT-2 robot and instead introduced a step where the plate was transferred to a shaker for digestion. However, Liang et al., in collaboration with evoSep, described PAC digestion on the OT-2 robot without shaking; instead, they briefly pipetted the solution up and down for 20 strokes.^25^

Phosphoenrichment using magnetic beads can, in principle, be conducted using the Platemaster P220, Alapaqua Magnum EX, and AB-0661 plates as described for PAC (Figure 7A), which is how we performed the procedure originally, but we would sometimes observe variability in contamination of phosphopeptides with unmodified peptides. This contamination in our opinion does not come from unmodified peptide retention on Zr-IMAC magnetic beads, but due to interactions with the plastic plate surfaces and also from liquid droplets retained by the plasticware. Therefore, it is crucial to transfer the beads from the original plate, where they were incubated with the peptide solution, to a new plate at least once. In our experience, the MagPin device is very efficient for this transfer and yields exceptionally reproducible enrichment results.

Another important component of the work described here is the introduction of self-packed Oasis HLB plates. Given the widespread use of this material in proteomics workflows, it will be very useful to the community to be able to manufacture these plates at a low cost and with a custom amount of the Oasis HLB material. A key aspect of this material that makes it reliable for desalting is its ability to be wetted by aqueous solutions. However, this property also means that the material retains liquid after sample loading and washing, which dilutes the elution buffer. By quantifying the exact extent of liquid retention (1.3 μL/mg of sorbent), we can achieve precise concentrations of ACN in the eluate. Additionally, we demonstrate that the concentration of ACN necessary to elute peptides is 40%, as opposed to 70% recommended by Waters. Unfortunately, lowering the ACN concentration to this level is ineffective at removing lipid contamination.

The reproducibility of our workflow can be compared with previously published automated and semiautomated sample-preparation methods, but such comparisons should be interpreted cautiously. Most publications report total analytical CVs, which reflect the combined variability from sample preparation, LC–MS analysis, and data analysis. In addition, CVs are inherently sample-type dependent; for example, biofluids typically exhibit higher variability due to their challenging dynamic range.^44^

Nevertheless, the current platform performs favorably relative to prior reports. For instance, the Van Eyk group reviewed existing sample-preparation strategies and concluded that clinical assays should achieve CVs <20%. In their own studies, they report CVs of 3–10% for spiked-in proteins, with sample preparation steps contributing 3–6%.^6^

The developers of SP3 automated their workflow on an Agilent Bravo system and reported median protein measurement CVs of 10–12%.^20^ These values are higher than those observed in our study, although their work relied on DDA, a method known to be inherently less reproducible than the DIA strategy implemented here.^22^

A recent report describing PAC automation on an Opentrons OT-2, using a three-protein mixture and DIA analysis, showed median CVs of ∼4% for *E. coli*, 5% for human, and 10% for yeast proteins.^23^ At the peptide level (without phosphoenrichment), they observed a median CV of 17–19%, which is comparable to the phospho-site-level CVs reported in our study.

An important consideration for future method-development studies focusing on innovations in sample preparation is the inclusion of pooled samples, as implemented in our work. This approach enables the separation of variability arising from sample preparation from that introduced by LC–MS analysis. In contrast, reporting only the total analytical CV—which conflates both sources of variation—is of limited value, particularly given the rapid evolution of proteomics LC–MS instrumentation.

## Conclusion

As mentioned earlier, we do not view the described workflow as a suitable alternative to fully automated liquid handlers, which are becoming increasingly reliable and versatile. Instead, the proposed methodology serves as an excellent stepping stone for laboratories that aim to achieve full automation. When developing a fully automated protocol, it is strongly recommended that the workflow be performed manually first. This step helps researchers understand the nuances of sample handling and identify potential sources of variability such as inadequate mixing, tip blockage, or sample losses during transfer. The devices presented here facilitate this process by exposing users to 96-well format tools, providing an effective bridge between manual and fully automated workflows.

## Supplementary Material













## Data Availability

Data are available via ProteomeXchange with identifier PXD069607.

## Notes

Figure 1—applicability of the Oasis HLB material for the removal of lipid contamination, Figure 2—robustness of direct injection chromatography for phosphoproteomics analysis of desalted and non-desalted samples, Figure 3—proportion of missed-cleavage peptides across varying pipetting and shaking durations in the protease solution, Figure 4—proportion of missed-cleavage peptides across varying pipetting durations, comparing conditions in which beads were retained in the sample versus removed after settling, and Figure 5—estimation of peptide recoveries across varying pipetting durations, comparing conditions in which beads were retained in the sample versus removed after settling (PDF)
Weights of empty tips and tips containing varying amounts of dry or wet Oasis HLB material (CSV)
Root-mean-square error (RMSE) and standard deviation of measured ratios in the three-proteome experiments­(XLSX)
Manufacturing of Oasis HLB self-packed plates (MP4)
Protein aggregation capture using Gilson Platemaster P220 (MP4)
Magnetic bead washes for phosphoproteomics using a Gilson Platemaster P220 pipettor and a VP Scientific 96-well Magnetic Pin Device (MP4)
