# Supplementary material for: High-Throughput Proteomics Sample Preparation Using a 96-Channel Pipettor and Magnetic Pin Device
Source: J Proteome Res. 2026 Feb 16;25(3):1647–61. doi: 10.1021/acs.jproteome.5c01020 (PMC12973302; doi:10.1021/acs.jproteome.5c01020)
Supplement: Supplementary file 1 [file pr5c01020_si_001.pdf]

Supporting Information

High-Throughput Proteomics Sample Preparation Using 96-Channel Pipettor and Magnetic Pin Device.

Georgia Roumelioti †[12], Alex Montoya†[12], Gemma L. M. Fisher[12], Eneko Pascual Navarro[12], Angela Woods[12], Jane Bennett[12], Naveenan Navaratnam[12], Oliver Gonzalez-Carvajal[12], Jodie Birch[12], Elizabeth Pyman[12], Sijia Yu[12], Aleksandra Gruevska[3], Luc-Alban Vuillemenot[12], Oleh Lushchak[12], Zoe Hall[3], Alexis R. Barr[12], Christian Speck[12], Santiago Vernia[12], William R. Scott[12], Jesus Gil[12], Luis Aragon[12], Louise Fets[12], David Carling[12] and Pavel V Shliaha[12]*

[1]MRC Laboratory of Medical Sciences (LMS), London, UK. W12 0HS

[2]Institute of Clinical Sciences, Imperial College London, Hammersmith Hospital Campus, London, UK. W12 0HS

3 Department of Metabolism, Digestion and Reproduction, Imperial College London, Hammersmith Hospital Campus, London, UK. W12 0NN

***pshliaha@ic.ac.uk**

Table of Contents

Supplementary Table 1 – Weights of Empty Tips and Tips Containing Varying Amounts of Dry or Wet Oasis HLB Material. (Supplementary_Table_1.csv file)

Supplementary Table 2 – Root mean square error (RMSE) and standard deviation (SD) of measured ratios in the three proteome experiments. (Supplementary_Table_2.xlsx file)

Supplementary Figure 1 - Applicability of Oasis HLB material for the Removal of Lipid Contamination

Supplementary Figure 2 - Robustness of Direct Injection Chromatography for Phosphoproteomics Analysis of Desalted and Non-Desalted Samples.

Supplementary Figure 3 - Proportion of missed-cleavage peptides across varying pipetting and shaking durations in the protease solution

Supplementary Figure 4 - Proportion of missed-cleavage peptides across varying pipetting durations, comparing conditions in which beads were retained in the sample versus removed after settling.

Supplementary Figure 5 - Estimation of peptide recoveries across varying pipetting durations, comparing conditions in which beads were retained in the sample versus removed after settling.

Supplementary Video 1 – Manufacturing of Oasis HLB self-packed plates. (Supplementary_Video_1_manufacturing_of_oasis_HLB_bead_plate.mp4 file)

Supplementary Video 2 – Protein Aggregation Capture Using Gilson Platemaster P220 (Supplementary_Video_2_PAC_procedure_execution.mp4 file)

Supplementary Video 3 – Magnetic Bead Washes for Phosphoproteomics using Gilson Platemaster P220 pipettor and VP Scientific 96-well Magnetic Pin Device (Supplementary_Video_3_magnetic_bead_wash_for_phosphoproteomics.mp4)

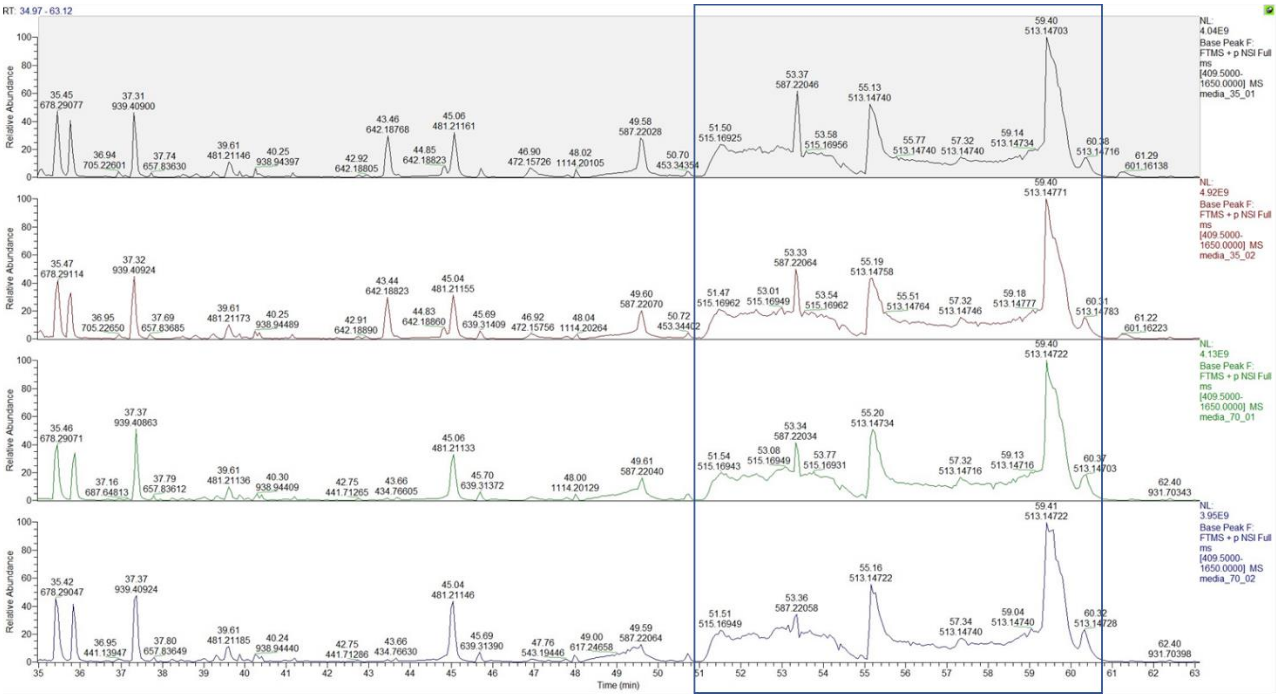

Supplementary Figure 1. Applicability of Oasis HLB material for the removal of lipid contamination. Lipid contamination after desalting was analyzed using conditioned media, with elution set at 35% (top two panels) and 70% ACN concentrations (bottom two panels). It is noted that there is no difference in intensity between the large lipid peaks eluting between 51 and 61 minutes.

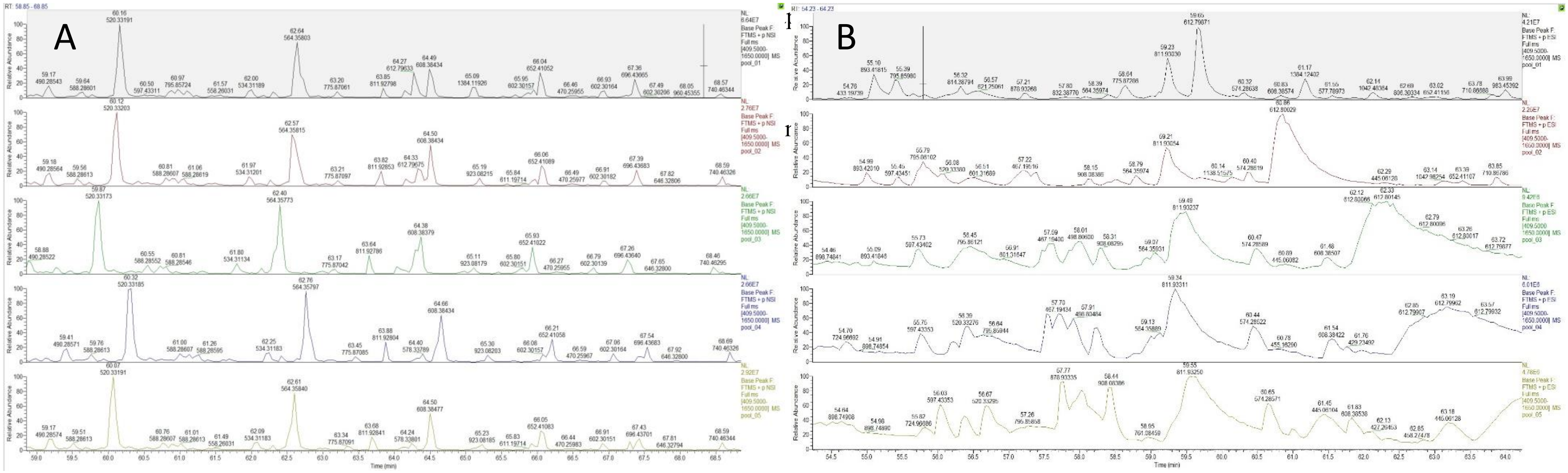

Supplementary Figure 2. Robustness of direct injection chromatography for phosphoproteomics analysis. A. For desalted samples. Shown are quality control pooled injections, occurring at six-sample intervals (i.e., injections # 1, 7, 13, etc.) B. For non-desalted samples. Shown are quality control pooled injections, occurring at six-sample intervals (i.e., injections 1, 7, 13, etc.)

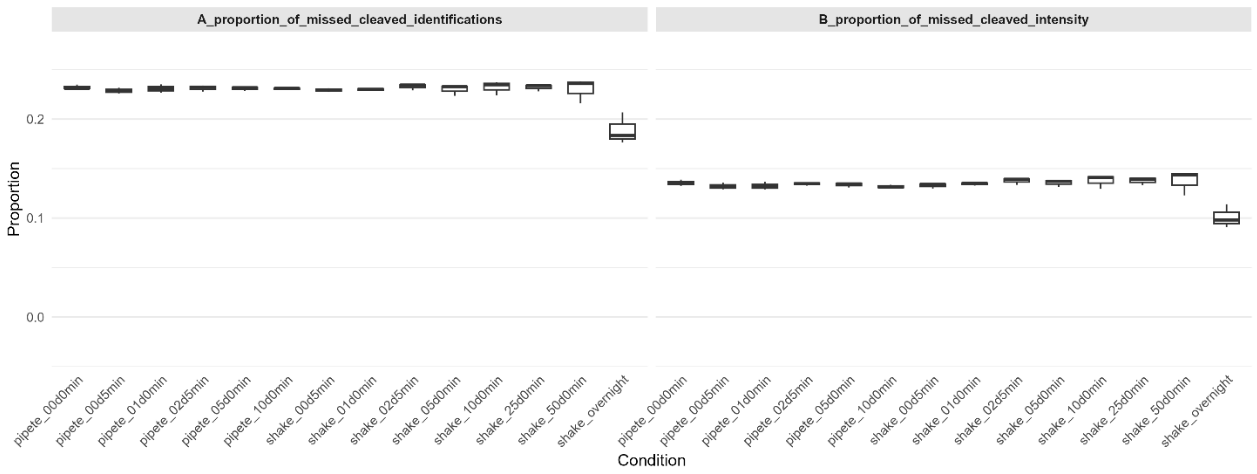

Supplementary Figure 3. Proportion of missed-cleaved peptides as a function of identification count (A) and their contribution to the overall signal (B) across varying pipetting and shaking times in the protease solution.

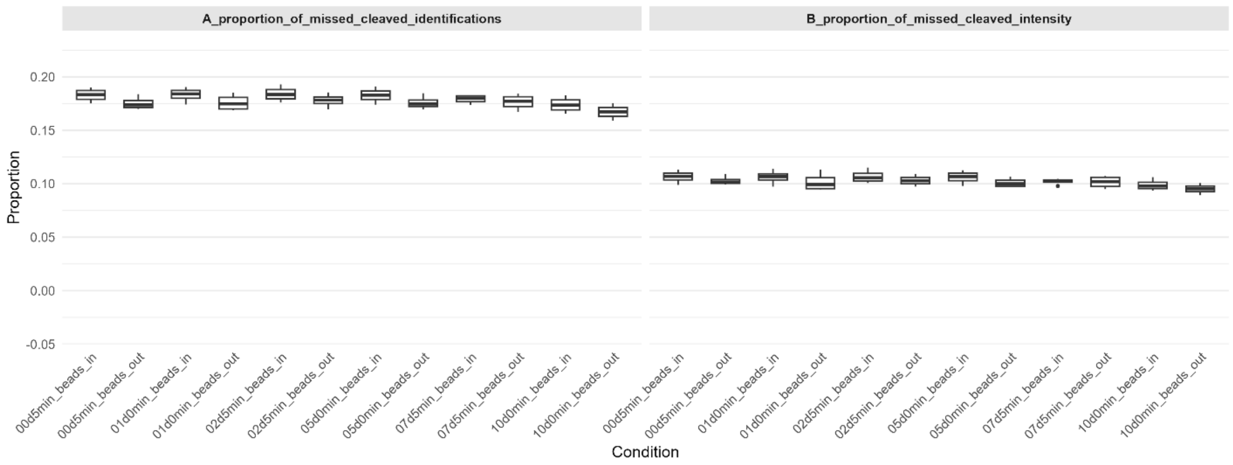

Supplementary Figure 4. Proportion of missed-cleaved peptides as a function of identification count (A) and their contribution to the overall signal (B) across varying pipetting times, comparing conditions where beads were left in the sample versus removed after settling.

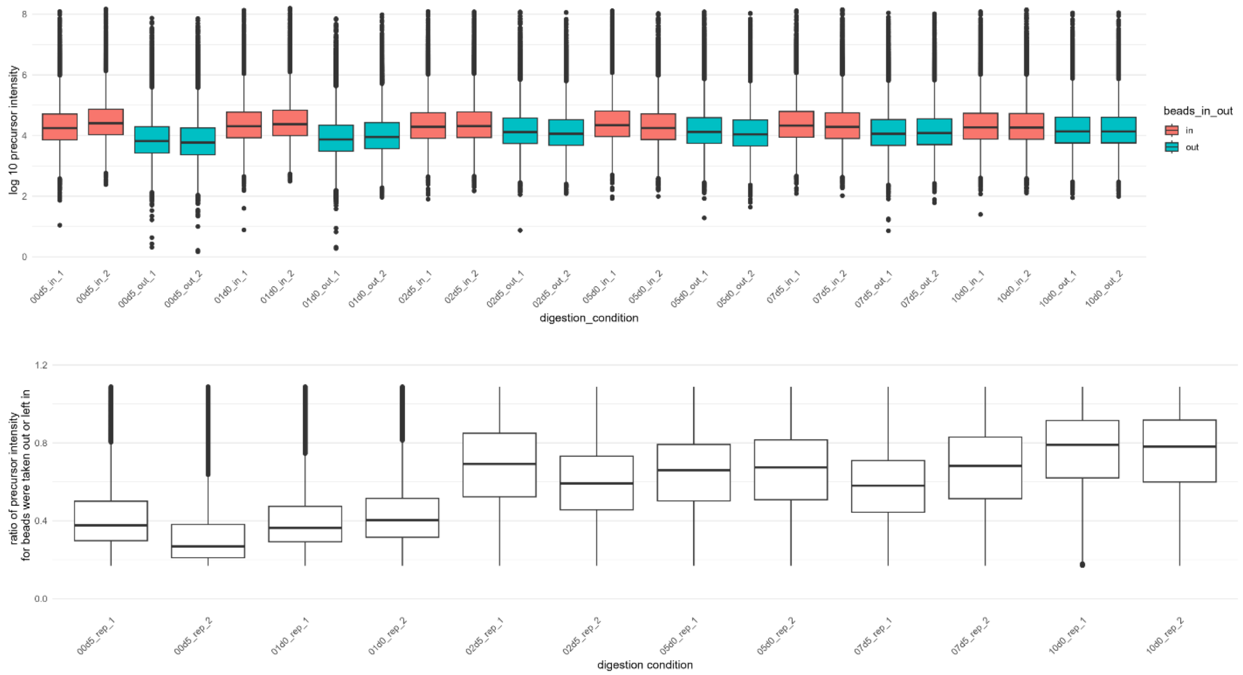

Supplementary Figure 5. Estimation of peptide recoveries for different digestion times and bead handling after settling. A. Log_{10} precursor intensities under various digestion conditions. B. Ratios of precursor intensities comparing samples where beads were removed versus samples where beads were retained during digestion.
